# Association of Smoking and Alcohol with Abdominal Aortic Calcification in the General Middle-Aged and Elderly Populations

**DOI:** 10.31083/RCM26087

**Published:** 2025-03-21

**Authors:** Yinze Ji, Naqiang Lv, Aimin Dang

**Affiliations:** ^1^Premium Care Center, Department of Cardiology, Fuwai Hospital, Chinese Academy of Medical Sciences & Peking Union Medical College, National Clinical Research Center for Cardiovascular Diseases, National Center for Cardiovascular Diseases, 100037 Beijing, China

**Keywords:** smoking, alcohol consumption, abdominal aortic calcification, abdominal aortic calcification score

## Abstract

**Background:**

Research results on the association between alcohol consumption and abdominal aortic calcification (AAC) has yielded inconsistent results. There is a paucity of evidence on the association of smoking and alcohol consumption with AAC in the general middle-aged and elderly population, including age subgroups. This study utilizes nationwide survey data to explore these associations.

**Methods:**

Data from middle-aged and elderly National Health and Nutrition Examination Survey (NHANES) 2013–2014 participants receiving dual X-ray absorptiometry were analyzed. AAC severity was assessed using a scoring system with a maximum value of 24. Presence of AAC was defined as an AAC score >0, and severe AAC as an AAC score ≥6. Binary logistic regression was employed for analyzing the association of smoking and alcohol consumption-related indices with the presence of AAC, while cumulative odds logistic regression explored their associations with severe AAC.

**Results:**

Data of 3135 participants were analyzed. Investigation in the entire population found that smoking history was linked to both AAC and severe AAC. In contrast, alcohol consumption history was not linked to AAC or severe AAC. After adjusting for confounders, the findings confirmed a significant association of smoking history with AAC and severe AAC. No significant associations were found for current alcohol consumption with either AAC or severe AAC. Compared with never smokers, former smokers and current smokers experienced increased AAC risk. Former smokers had a significantly lower AAC risk compared to current smokers. Compared with never alcohol consumers, neither former nor current alcohol consumers experienced a different AAC risk. No difference in AAC risk was found between former and current alcohol consumers. Individuals consuming more than 2 drinks of alcohol per day suffered from a significant increase in risk of AAC. Subgroup analyses found elderly ever and current smokers suffered from a significantly elevated AAC risk, as did middle-aged ever smokers. Elderly ever and current alcohol consumers also experienced increased risk of AAC.

**Conclusions:**

Smoking history is significantly associated with both AAC and severe AAC. The cardiovascular benefits associated with smoking cessation primarily manifest as reduction in risk of AAC presence rather than severe AAC. Elderly smokers are exposed to a greater risk of AAC. In contrast, alcohol consumption shows no association with severe AAC. Alcohol consumption is not associated with AAC except in heavy drinking and elderly subpopulations.

## 1. Introduction

Abdominal aortic calcification (AAC) is a common subtype of vascular calcification acknowledged not only as a marker of atherosclerosis [1, 2] but also as a predictor of poor cardiovascular prognosis. Studies have linked AAC to increased risks of all-cause mortality [3, 4], cardiovascular mortality [4], as well as both fatal and nonfatal cardiovascular events [3, 5]. Furthermore, in dialysis patients, AAC correlates with an increase in left ventricular mass [6]. Given its association with these clinically significant outcomes, analyzing risk factors for AAC is crucial for its prevention and the mitigation of adverse cardiovascular events.

Smoking is an acknowledged risk factor of atherosclerosis. Pro-inflammatory and endothelial-damaging components in cigarette smoke have been demonstrated to promote the development of this endothelial injury and atherosclerosis [7]. Despite the well-established link between atherosclerosis and vascular calcification [8], research into the association between smoking and AAC has been predominantly limited to populations with certain characteristics, such as males or individuals afflicted by chronic kidney disease (CKD). For instance, Jung *et al*. [9] found smoking-related indices like cumulative smoking duration and cumulative smoking amount correlated with AAC in a cohort of 218 middle-aged and elderly men. Similarly, Lioufas *et al*. [10] also found smoking to be associated with AAC in CKD patients. Furthermore, Pham *et al*. [11] found that, after adjusting for age and other cardiovascular risk factors, current smokers exhibited an increased risk of AAC progression compared to non-smokers. Zhang *et al*. [12] found that populations with a smoking history faced an increase in risk of AAC exacerbation. These evidence underscores the need for further investigation into smoking’s role in AAC development across broader demographic groups.

Research on the relationship between alcohol consumption and AAC has yielded inconsistent results. Forbang *et al*. [13] found that alcohol consumption was correlated with an increase in the natural logarithm of AAC volume. In contrast, Wu [14] found no significant association between alcohol consumption history and AAC in a population of patients with CKD Stage 3–5. In a more recent study examining a cross-sectional sample of the general United States (U.S.) population no significant differences in AAC prevalence among never, former, mild, moderate and heavy alcohol consumers were found [15]. These divergent outcomes highlight the complexity of the relationship between alcohol consumption and AAC development, suggesting that additional research is needed to delineate the underlying mechanisms and potential moderating factors.

Despite the well-documented link between smoking and atherosclerosis, the specific association between smoking and AAC remains underexplored, particularly in the general middle-aged and elderly population. Studies utilizing nationwide survey data for the examination of this association are notably scarce. Similarly, existent research on the link between alcohol consumption and AAC is limited and yields inconsistent results. Consequently, there is a significant need to undertake comprehensive studies using nationwide survey data to investigate these associations. Such research could provide crucial insights into the prevention of AAC and its associated adverse events.

## 2. Materials and Methods

### 2.1 Study Design

This study utilized data of the National Health and Nutrition Examination Survey (NHANES), a nationwide survey of United States residents that has operated continuously since 1999. In 2013 and 2014, a subgroup of middle-aged and elderly participants underwent dual energy X-ray absorptiometry to assess AAC and other medical conditions. The current study abides by the Declaration of Helsinki throughout the entire course and was approved by Ethics Committee of Fuwai Hospital, Chinese Academy of Medical Sciences and Peking Union Medical College (Approval No.: 2021-1461). As per consensus on informed consent [16], informed consent was not feasible since this study utilized data of de-identified individuals from a database and did not involve any personally identifiable information.

### 2.2 Study Population

Participants of NHANES 2013-2014 who received dual energy X-ray absorptiometry and had AAC severity measured by AAC score (see below for more details) were included in the study. Participants with insufficient information were excluded from the study.

### 2.3 Data Collection

NHANES collected a series of information on the participants, including demographic, laboratory, and imaging data. More specifically, demographic data (e.g., age, sex, race), tobacco and alcohol consumption history, history of comorbidities (e.g., hypertension, coronary heart disease), and medication history including use of aspirin and statins were collected via questionnaires. Cardiovascular medication was defined as the composite of anti-platelet, lipid-lowering, anti-hypertensive agents, oral hypoglycemics, and insulin in this study. Participants using any kind of the aforementioned cardiovascular medications were defined as receiving cardiovascular medical therapy while those not using cardiovascular medications from the list were defined as not receiving cardiovascular medical therapy. Venous blood was drawn from participants for biomarker analyses. Lateral spine radiograph obtained via dual energy X-ray absorptiometry was used for assessment of AAC, while AAC score was used for quantifying its severity. Briefly, the anterior and posterior abdominal aortic walls were partitioned into four segments corresponding to the position of the first to the fourth lumbar vertebrae respectively. Determination of AAC severity was conducted for each segment via the following scoring method: AAC was scored as “0” if there was no calcification; “1” if one-third or less than one-third of the aortic wall in that segment was calcified; “2” if more than one-third but less than two-thirds was calcified; or “3” if more than two-thirds was calcified. With a probable range of 0 to 24, AAC score of the participant was the sum of scores in each segment [17].

### 2.4 Definition of Presence of AAC and Severe AAC

The presence of AAC was defined by an AAC score above 0. Following the definition adopted by prior studies [18, 19, 20], severe AAC was defined as AAC score not lower than 6.

### 2.5 Definition of Smoking and Alcohol Consumption-Related Indices

Information regarding smoking and alcohol consumption was collected via survey questionnaires, where in most cases participants or their proxies were asked to choose among a variety of choices whose exact wordings had been written out in advance. Given the choices offered in questionnaires and potential answers by respondents, this study defined smoking and alcohol consumption-related indices. Individuals with smoking history were those who ever smoked at least 100 cigarettes. Individuals without smoking history were those that denied ever smoking at least 100 cigarettes. Current smokers were those with at least one of the following conditions: (1) current cigarette smoking frequency was reported as “everyday” or “some days”; (2) smoked tobacco or used smokeless tobacco in the past 5 days. Current non-smokers were those with all the following conditions: (1) current cigarette smoking frequency was reported as “not at all”; (2) denied smoking tobacco or using smokeless tobacco in the past 5 days.

Individuals with alcohol consumption history were those with at least one of the following conditions: (1) had consumed 12 alcohol drinks per year (a drink of alcohol was defined as 12 ounces of beer, a 5-ounce glass of wine or 1.5 ounces of liquor); (2) had consumed at least 12 alcohol drinks in their life; (3) existed a period of time in life that the participant drank 4 or 5 drinks of alcoholic beverage almost every day. Individuals without alcohol consumption history were those denied drinking at least 12 alcohol drinks in their lifetime. Current alcohol consumers were those with at least one of the following conditions: (1) frequency of alcohol consumption in the past 12 months (the number of times consuming any type of alcoholic beverage measured in weeks, months or years) exceeding zero; (2) consumed alcohol in the period of time between last food intake to drawing venous blood. Individuals not currently consuming alcohol were those that did not consume alcohol in the past 12 months.

### 2.6 Statistical Analyses

Data of NHANES were collected via complex sampling from a finite population instead of simple random sampling from an infinite population, an issue that should be taken into account in the analytic process. In other words, statistical methods tailored for complex survey data instead of the “generic” ones that, in many cases, implicitly assume the data came from simple random sampling should be applied. Briefly, for description of clinical characteristics of the entire studied population, the normalities of continuous variables were assessed via the quartet of modified Cramér-von Mises tests tailored for complex survey data, histograms, probability-probability plots (P-P plots), and quantile-quantile plots (Q-Q plots). Given that when data are collected in complex surveys, the means and quantiles follow *t* distributions and can hence be pooled via Rubin’s rules during multiple imputation, central and dispersion tendencies of continuous variables were reported in mean (1st quartile, 3rd quartile) for variables following normal distributions and median (1st quartile, 3rd quartile) for non-normal variables. Given the practice of conducting complex survey data analysis adopted by other medical research papers [21], percentages were reported for categorical variables. Linear regression was used for testing statistical significances of differences of continuous variables across AAC groups and for the adjustment of confounders in inter-group mean comparisons. Rao-Scott χ^2^ tests were used for inter-group comparisons of categorical variables. Binary logistic regression models for complex survey data were used to analyze the association between smoking and alcohol consumption with presence of AAC. Cumulative odds logistic regression models for complex survey data were used for analyzing the relationship of smoking and alcohol consumption with severe AAC. The jackknife method was adopted for variance estimation in the entire analytic process. Pseudo-maximum likelihood estimation was employed for computing the regression coefficients of the logistic regression models in the model fitting process, which was followed by statistical diagnostics of models tailored for complex survey data, a field that Lewis described as being “in its nascent stage” [22]. In fact, statistical methods tailored for finite population sampling have been, as Thompson [23] commented, “long regarded as lying outside the mainstream of statistical inference”, causing a paucity of methods available for researchers in the medical profession who attempt to elaborate the versatility of information that can be mined from the data to the greatest extent. Given the collection of available, suitable and hence retrieved statistical methods at the time of this writing, a goodness-of-fit test proposed by Archer and Lemeshow [24] (referred to as “Archer-Lemeshow test” hereafter), a variant of the commonly applied Hosmer-Lemeshow test popular in assessing goodness-of-fit in binary logistic regression models for data not collected from complex surveys, was employed for assessing goodness-of-fit of binary logistic regression models tailored for complex survey data. Multiple imputation was employed for addressing missing data. Factoring the complex survey nature of NHANES data in, fully conditional specification (FCS) logistic regression was used for imputation of categorical variables while FCS predictive mean matching (PMM) method was employed for imputation of continuous ones. Results generated on each imputed dataset were pooled via Rubin’s rules if the variable to be pooled followed a normal or *t*-distribution, a prerequisite of applying Rubin’s rules [25]. For variables following χ^2^ distributions, the D2 method [26] or the median *p *value method (applied where appropriate) was employed to pool the results. Variables following *F* distributions were pooled by a method proposed by Chaurasia [27]. To be more specific, a modification of the transformation of random variables from the *F* into the beta-distribution based upon the one originally proposed by Hodgson [28], the method proposed and suggested for further use in Chaurasia’s paper, was applied. The entire analytical process was conducted on Statistical Analysis System (SAS) version 9.4 TS1M5 (SAS Institute Inc., Cary, NC, USA). *p* values < 0.05 were considered statistically significant.

## 3. Results

### 3.1 Clinical Characteristics of the Population

A total of 10,175 individuals participated NHANES 2013–2014, with 3135 meeting the specified inclusion and exclusion criteria for our analyses. Table 1 summarizes clinical characteristics of participants across three AAC groups, as well as results of statistical tests for inter-group comparisons. Overall, many variables were significantly different among groups, with increasing age, diabetes diagnosis, and smoking history correlating with higher AAC scores. Concurrently, a decline in renal function, as measured by the estimated glomerular filtration rate (eGFR), was also observed with increasing AAC. Differences in the proportion of current alcohol drinkers also varied significantly among the three groups. No significant difference was found with respect to proportions of current smokers among the three groups. Results relevant to the association of smoking history, current smoking and AAC suggest that the risk of AAC for former smokers may remain elevated compared to that of never smokers. In addition, the association of alcohol consumption and AAC appeared to be time-dependent, which was exemplified by a decline in proportion of participants with ACC score ≥6 among current alcohol consumers.

**Table 1.** **Clinical characteristics of population by AAC scores**.

Variable	AAC score = 0 (2191 participants)	0 < AAC score < 6 (603 participants)	AAC score ≥6 (341 participants)	*p* value
Age (years)	54.89 (45.67, 61.66)	60.14 (50.33, 68.62)	70.58 (63.98, 79.10)	<0.001
Male sex (%)	47.88	50.73	44.22	0.372
Hypertension (%)	41.43	55.86	75.58	<0.001
Diabetes (%)	15.55	19.26	35.44	<0.001
Dyslipidemia (%)	83.83	83.78	87.67	0.386
CHD (%)	3.16	4.92	17.14	<0.001
Heart failure (%)	1.87	2.76	9.07	<0.001
Gout (%)	5.19	6.93	6.35	0.436
Myocardial infarction (%)	2.76	5.83	14.55	<0.001
Alcohol consumption history (%)	86.22	87.69	87.70	0.878
Current alcohol consumption (%)	70.87	71.67	59.50	0.039
Smoking history (%)	42.24	53.34	59.78	<0.001
Current smoking (%)	47.06	50.05	41.51	0.054
Aspirin (%)	19.09	31.93	55.07	<0.001
Lipid-lowering agents (%)	23.61	31.53	52.27	<0.001
Anti-hypertensive agents (%)	31.23	45.89	70.15	<0.001
Oral hypoglycemics (%)	8.79	9.82	19.21	<0.001
Insulin (%)	2.66	3.33	8.03	<0.001
Fasting HDL-C (mmol/L)	1.41 (1.08, 1.66)	1.35 (1.06, 1.59)	1.41 (1.09, 1.68)	0.039
Fasting LDL-C (mmol/L)	3.05 (2.26, 3.66)	2.97 (2.14, 3.61)	2.75 (1.98, 3.33)	0.014
Fasting triglyceride (mmol/L)	1.34 (0.80, 2.00)	1.43 (1.00, 2.09)	1.45 (0.95, 2.02)	0.091
Fasting blood glucose (mmol/L)	5.50 (5.16, 6.07)	5.61 (5.24, 6.34)	5.79 (5.28, 7.02)	0.001
Glycohemoglobin (%)	5.44 (5.21, 5.75)	5.53 (5.27, 5.93)	5.80 (5.43, 6.32)	<0.001
eGFR mL/(min × 1.73 m^2^)	85.49 (73.75, 98.53)	81.95 (70.12, 96.03)	68.07 (52.80, 84.12)	<0.001

Notes: Continuous variables following normal distributions were reported as mean (1st quartile, 3rd quartile). In contrast, variables not following a normal distribution were reported as median (1st quartile, 3rd quartile). For categorical variables, proportion was reported. Linear regression analysis was used for inter-group comparisons of continuous variables while the Rao-Scott χ^2^ test was employed to test the significance of inter-group differences of categorical variables. Abbreviations: CHD, coronary heart disease; eGFR, estimated glomerular filtration rate; HDL-C, high-density lipoprotein cholesterol; LDL-C, low-density lipoprotein cholesterol; AAC, abdominal aortic calcification. “Aspirin”, “Lipid-lowering agents”, “Anti-hypertensive agents”, “Oral hypoglycemics”, “Insulin” refer to usage of the medications.

### 3.2 Comparisons of Risk Factors Between Participants with and Without Cardiovascular Medications

To further elucidate the clinical characteristics of the examined population, participants were categorized based on whether or not they received cardiovascular medications, as outlined in the “Data collection” section. The comparative analysis between these two groups with respect to the presence or severity of risk factors are summarized in Table 2. The results indicate a higher burden of risk factors among those receiving cardiovascular medical therapy, as evidenced by a greater proportion of hypertensive, diabetic, and ever smoking participants. Additionally, this group displayed significantly older age, higher body mass index (BMI), reduced renal function, and poorer glycemic control, as measured by fasting glucose and glycohemoglobin levels, compared to those not receiving medical therapy. Despite these challenges, the analysis also highlighted better control of certain risk factors in the medical therapy group, including a significantly lower proportion of current smokers and reduced levels of low-density lipoprotein cholesterol (LDL-C).

**Table 2.** **Differences in risk factors between participants receiving or not receiving cardiovascular medications**.

Variable	No medication (1475 sampled individuals)	With medication (1660 sampled individuals)	*p* value
Age (years)	51.98 (43.93, 56.94)	62.58 (53.48, 70.90)	<0.001
Male sex (%)	48.28	47.90	0.767
Hypertension (%)	15.69	78.07	<0.001
Diabetes (%)	4.80	31.02	<0.001
Dyslipidemia (%)	83.96	85.10	0.596
Alcohol consumption history (%)	86.26	86.73	0.627
Current alcohol consumption (%)	73.94	66.56	0.018
Smoking history (%)	41.53	50.40	<0.001
Current smoking (%)	36.34	29.44	0.042
Fasting triglyceride (mmol/L)	1.29 (0.79, 1.94)	1.44 (0.91, 2.08)	0.045
Fasting HDL-C (mmol/L)	1.43 (1.10, 1.70)	1.37 (1.06, 1.60)	0.052
Fasting LDL-C (mmol/L)	3.13 (2.40, 3.72)	2.87 (2.08, 3.48)	0.003
Fasting glucose (mmol/L)	5.39 (5.08, 5.82)	5.77 (5.25, 6.76)	0.000
Glycohemoglobin (%)	5.35 (5.15, 5.60)	5.66 (5.36, 6.14)	<0.001
eGFR mL/(min × 1.73 m^2^)	89.38 (78.91, 101.52)	77.12 (64.56, 91.78)	<0.001

Notes: Participants were classified as receiving cardiovascular medications if they used any anti-platelet, lipid-lowering, anti-hypertensive agents, oral hypoglycemics, or insulin. Conversely, those not using any of these cardiovascular medications were defined as not receiving cardiovascular medications. Abbreviations: eGFR, estimated glomerular filtration rate; HDL-C, high-density lipoprotein cholesterol; LDL-C, low-density lipoprotein cholesterol.

### 3.3 Univariable Analyses of the Association Between Smoking History and the Presence of AAC or Severe AAC

Univariable analyses were conducted to examine the association between smoking-related indices and AAC. The findings indicated a significant association between a history of smoking and presence of AAC (odds ratio (OR) = 1.70, 95% confidence interval (CI): 1.30–2.22; *p* < 0.001), while current smoking showed no association with AAC (Table 3). The Archer-Lemeshow test confirmed the model’s good fit. Parallel results were found for severe AAC, where a significant association was found for smoking history (OR = 1.72, 95% CI: 1.33–2.21; *p *= 0.001), but not with current smoking (Table 4).

**Table 3.** **Univariable analyses of smoking and AAC**.

Variable	AAC score = 0 (2191 participants)	AAC score >0 (947 participants)	Regression coefficient	OR (95% CI)	*p* value	*p* value for GOF
Smoking history	42.89%	54.12%	0.53	1.70 (1.30, 2.22)	0.001	0.091
Current smoking	49.31%	45.44%	0.02	1.02 (0.11, 9.60)	0.958	0.093

Abbreviations: AAC, abdominal aortic calcification; CI, confidence interval; GOF, goodness-of-fit; OR, odds ratio.

**Table 4.** **Univariable analyses of smoking and severe AAC**.

Variable	AAC score <6 (2799 participants)	AAC score ≥6 (341 participants)	Regression coefficient	OR (95% CI)	*p* value
Smoking history	44.71%	59.11%	0.54	1.72 (1.33, 2.21)	0.001
Current smoking	49.24%	39.90%	–0.02	0.98 (0.06, 16.98)	0.965

Abbreviations: AAC, abdominal aortic calcification; CI, confidence interval; OR, odds ratio.

### 3.4 Univariable and Multivariable Comparisons of AAC Risk Among Never, Former and Current Smokers

Univariable analyses (i.e., analyses not adjusted for confounders) revealed that both former (OR = 1.68, 95% CI: 1.38–2.04; *p* < 0.001) and current (OR = 1.69, 95% CI: 1.16–2.45; *p* = 0.009) smokers exhibited a significantly higher risk of AAC compared to never smokers, as shown in Table 5. Interestingly, there was no significant difference in AAC risk between former and current smokers, suggesting a persistently elevated risk regardless of smoking cessation.

**Table 5.** **Univariable analysis of smoking and AAC risk among never, former and current smokers**.

Population	Regression coefficient	OR (95% CI)	*p* value	*p* value for GOF
Never smokers	Reference group			0.530
Former smokers	0.52	1.68 (1.38, 2.04)	<0.001	
Current smokers	0.52	1.69 (1.16, 2.45)	0.009	
Current smokers	Reference group			
Former smokers	–0.01	0.99 (0.72, 1.37)	0.961	

Abbreviations: AAC, abdominal aortic calcification; CI, confidence interval; GOF, goodness-of-fit; OR, odds ratio.

In the multivariable analyses (i.e., analyses adjusted for confounders), the differences in AAC risk between smoking groups became more pronounced, as detailed in Table 6. After adjusting for confounders, both former (OR = 1.30, 95% CI: 1.07–1.59; *p* = 0.013) and current smokers (OR = 2.36, 95% CI: 1.54–3.61; *p* < 0.001) still demonstrated an elevated risk of AAC compared to never smokers. However, the risk in former smokers was significantly lower than in current smokers (OR = 0.55, 95% CI: 0.40–0.77; *p* = 0.002), suggesting a notably reduced risk.

**Table 6.** **Multivariate analysis of smoking history and AAC risk among never, former and current smokers**.

Population	Regression coefficient^*^	OR^*^ (95% CI)^*^	*p* value^*^	*p* value for GOF
Never smokers	Reference group			0.593
Former smokers	0.27	1.30 (1.07, 1.59)	0.013	
Current smokers	0.86	2.36 (1.54, 3.61)	0.001	
Current smokers	Reference group			
Former smokers	–0.59	0.55 (0.40, 0.77)	0.002	

Note: ^*^adjusting for sex, elderly age (defined as age ≥65 years old), current alcohol consumption, diabetes, hypertension, coronary heart disease, fasting low-density lipoprotein cholesterol and usage of lipid-lowering agents. Abbreviation: GOF, goodness-of-fit; AAC, abdominal aortic calcification; CI, confidence interval; OR, odds ratio.

### 3.5 Smoking-Associated Risk of AAC Between Different Middle-Aged and Elderly Populations

To examine the association between AAC and smoking more closely, we analyzed the risks related to smoking-history and current smoking behaviors across different age subgroups, as reported in Tables 7,8. This began by comparing middle aged never smokers to ever smokers in middle aged and elderly populations. Elderly people who ever smoked experienced a significantly higher risk of AAC presence compared to their middle-aged never smoker counterparts (OR = 5.07, 95% CI: 3.24–7.94; *p* < 0.001). Interestingly, while the risk for middle-aged ever smokers was also elevated (OR = 1.93, 95% CI: 1.37–2.70; *p *= 0.001) compared with middle-aged never smokers, no significant difference in the smoking-history associated increment in risk of AAC between the middle-aged and elderly populations was found.

**Table 7.** **Comparisons of smoking history-associated AAC risk between middle-aged and elderly populations**.

Population	Regression coefficient^*^	OR^*^ (95% CI)^*^	*p* value^*^	*p* value for interaction^*^	*p* value for GOF
Middle-aged never smokers	Reference group			0.087	0.705
Middle-aged ever smokers	0.66	1.93 (1.37, 2.70)	0.001		
Elderly ever smokers	1.62	5.07 (3.24, 7.94)	<0.001		

Note: ^*^adjusting for sex, diabetes, hypertension, coronary heart disease, fasting low-density lipoprotein cholesterol and usage of lipid-lowering agents. Abbreviations: CI, confidence interval; GOF, goodness-of-fit; OR, odds ratio; AAC, abdominal aortic calcification.

**Table 8.** **Comparisons of current smoking-associated AAC risk between middle-aged and elderly populations**.

Population	Regression coefficient^*^	OR^*^ (95% CI)^*^	*p* value^*^	*p* value for interaction^*^	*p* value for GOF
Middle-aged current non-smokers	Reference group			0.580	0.489
Middle-aged current smokers	0.41	1.51 (0.28, 8.26)	0.423		
Elderly current smokers	1.42	4.16 (2.01, 8.61)	0.002		

Note: ^*^adjusting for sex, diabetes, hypertension, coronary heart disease, fasting low-density lipoprotein cholesterol and usage of lipid-lowering agents. Abbreviations: CI, confidence interval; GOF, goodness-of-fit; OR, odds ratio; AAC, abdominal aortic calcification.

We next compared middle-aged current non-smokers to current smokers in middle aged and elderly populations (Table 8). Elderly current smokers faced a substantially increased risk of AAC compared to middle-aged current non-smokers (OR = 4.16, 95% CI: 2.01–8.61; *p *= 0.002). However, the difference in increment of AAC risk between middle-aged current smokers and their elderly counterparts with respect to middle-aged current non-smokers is not statistically significant, suggesting that the associations of current smoking might be consistent across these age groups. Both tables confirmed the models’ good fit, validating the reliability of the results.

### 3.6 Univariable Analyses of Alcohol Consumption and Presence of AAC or Severe AAC

Univariable analyses of the association of alcohol consumption and AAC are detailed in Tables 9,10. Both tables examined the association of alcohol consumption history and current alcohol consumption with AAC- presence of and severe respectively. The results revealed no significant correlations between alcohol consumption (both ever and current) and the presence or severity of AAC. Both models demonstrated in the table well fit the data.

**Table 9.** **Univariable analyses of alcohol consumption and AAC**.

Variable	AAC score = 0 (2191 participants)	AAC score >0 (947 participants)	Regression coefficient	OR (95% CI)	*p* value	*p* value for GOF
Alcohol consumption history	84.54%	85.92%	0.13	1.14 (0.82, 1.57)	0.401	0.124
Current alcohol consumption	65.30%	61.75%	–0.15	0.86 (0.66, 1.12)	0.244	0.754

Abbreviations: AAC, abdominal aortic calcification; CI, confidence interval; GOF, goodness-of-fit; OR, odds ratio.

**Table 10.** **Univariable analyses of alcohol consumption and severe AAC**.

Variable	AAC score <6 (2799 participants)	AAC score ≥6 (341 participants)	Regression coefficient	OR (95% CI)	*p* value
Alcohol consumption history	84.68%	87.23%	0.12	1.13 (0.81, 1.57)	0.426
Current alcohol consumption	65.21%	56.23%	–0.20	0.82 (0.64, 1.06)	0.116

Abbreviations: AAC, abdominal aortic calcification; CI, confidence interval; OR, odds ratio.

### 3.7 AAC Risk Among Never, Former and Current Alcohol Consumers

Risks of presence of AAC was also examined among never, former and current alcohol consumers. Univariable analyses (Table 11) revealed that former alcohol consumers experienced an insignificant trend towards increased AAC risk compared with never alcohol consumers (OR = 1.39, 95% CI: 0.98–1.97; *p *= 0.061). Current alcohol consumers, however, did not show a significantly different risk compared to never alcohol consumers (OR = 1.02, 95% CI: 0.73–1.43; *p* = 0.896). Interestingly, when comparing former to current alcohol consumers, the former showed a significantly higher risk of AAC (OR = 1.36, 95% CI: 1.03–1.80; *p* = 0.032). As is the case with comparisons of risks among populations with different smoking status, results of this section need to be further verified by adjustment of confounders.

**Table 11.** **Univariable analyses of alcohol consumption and AAC risk among never, former and current alcohol consumers**.

Population	Regression coefficient	OR (95% CI)	*p* value	*p* value for GOF
Never alcohol consumers	Reference group			0.915
Former alcohol consumers	0.33	1.39 (0.98, 1.97)	0.061	
Current alcohol consumers	0.02	1.02 (0.73, 1.43)	0.896	
Current alcohol consumers	Reference group			
Former alcohol consumers	0.31	1.36 (1.03, 1.80)	0.032	

Abbreviations: CI, confidence interval; GOF, goodness-of-fit; OR, odds ratio; AAC, abdominal aortic calcification.

After adjusting for various confounders, the multivariable analyses (Table 12) indicated that neither former (OR = 0.99, 95% CI: 0.69–1.42; *p* = 0.946) nor current (OR = 1.06, 95% CI: 0.81–1.37; *p* = 0.652) alcohol consumers experienced a significantly different risk of AAC compared to never alcohol consumers. Moreover, the risk did not significantly differ between former and current alcohol consumers (OR = 0.94, 95% CI: 0.67–1.30; *p* = 0.669) after confounder adjustment.

**Table 12.** **Multivariable analyses of alcohol consumption and AAC risk among never, former and current alcohol consumers**.

Population	Regression coefficient^*^	OR^*^ (95% CI)^*^	*p* value^*^	*p* value for GOF
Never alcohol consumers	Reference group			0.252
Former alcohol consumers	–0.01	0.99 (0.69, 1.42)	0.946	
Current alcohol consumers	0.06	1.06 (0.81, 1.37)	0.652	
Current alcohol consumers	Reference group			
Former alcohol consumers	–0.07	0.94 (0.67, 1.30)	0.669	

Note: ^*^adjusting for sex, age (defined as age ≥65 years old), smoking history, diabetes, hypertension, coronary heart disease, fasting low-density lipoprotein cholesterol and usage of lipid-lowering agents. Abbreviations: AAC, abdominal aortic calcification; CI, confidence interval; OR, odds ratio; GOF, goodness-of-fit.

### 3.8 Univariable and Multivariable Comparisons of AAC Risk among Populations with Different Amount of Alcohol Intake

In the following exploration of the association between alcohol intake and the risk of AAC, we partitioned current alcohol consumers into groups based on the amount and frequency of alcohol intake per day over the past 12 months. Univariable analyses indicated that former alcohol consumers experienced a marginally statistically significant increase in AAC risk (OR = 1.41, 95% CI: 1.00–1.99; *p* = 0.048). However, among current alcohol consumers, the risk did not significantly differ across varying consumption levels (Table 13).

**Table 13.** **Univariable comparisons of AAC risk among populations consuming different amount of alcohol**.

Population	Regression coefficient	OR (95% CI)	*p* value	*p* value for GOF
Never alcohol consumers	Reference group			0.296
Former alcohol consumers	0.35	1.41 (1.00, 1.99)	0.048	
1 drink per day	0.01	1.01 (0.69, 1.48)	0.967	
2 drinks per day	–0.01	0.99 (0.66, 1.49)	0.956	
>2 drinks per day	0.15	1.17 (0.78, 1.75)	0.426	

Note: “1 drink per day” refers to consuming 1 drink of alcohol per day in the past 12 months. The same time frame is applied to subjects consuming 2 drinks per day and more than 2 drinks per day, who are denoted as “2 drinks per day” and “>2 drinks per day” in the table. Abbreviations: CI, confidence interval; GOF, goodness-of-fit; OR, odds ratio; AAC, abdominal aortic calcification.

Building upon this initial analysis, a multivariable analysis (Table 14) further dissected these associations. Notably, the results revealed that for all groups except those consuming more than two drinks per day, AAC risk was not statistically significantly different compared to never alcohol consumers. However, individuals consuming more than two drinks per day experienced a significantly elevated risk (OR = 1.53, 95% CI: 1.11–2.12; *p* = 0.013). This finding underscores the importance of moderating alcohol intake to potentially reduce the risk of AAC and its associated adverse clinical outcomes. These results highlight the complex relationship between alcohol consumption and AAC risk, suggesting that higher levels of consumption may indeed contribute to atherosclerosis.

**Table 14.** **Multivariable comparisons of AAC risk among populations consuming different amount of alcohol**.

Population	Regression coefficient^*^	OR^*^ (95% CI)^*^	*p* value^*^	*p* value for GOF
Never alcohol consumers	Reference group			0.548
Former alcohol consumers	0.04	1.05 (0.73, 1.49)	0.792	
1 drink per day	–0.05	0.95 (0.68, 1.32)	0.738	
2 drinks per day	0.11	1.11 (0.84, 1.48)	0.432	
>2 drinks per day	0.43	1.53 (1.11, 2.12)	0.013	

Note: ^*^adjusting for sex, diabetes, hypertension, smoking history, coronary heart disease, fasting low-density lipoprotein cholesterol and usage of lipid-lowering agents. “1 drink per day” refers to consuming 1 drink of alcohol per day in the past 12 months. The same time frame is applied to subjects consuming 2 drinks per day and more than 2 drinks per day, who are denoted as “2 drinks per day” and “>2 drinks per day” in the table. Abbreviations: CI, confidence interval; GOF, goodness-of-fit; OR, odds ratio; AAC, abdominal aortic calcification.

### 3.9 Alcohol Consumption-Associated Risk of AAC between Different Middle-Aged and Elderly Populations

Utilizing results from a well fit logistic regression model, we discovered that elderly ever alcohol consumers exhibited a significantly increased risk of AAC (OR = 3.78, 95% CI: 2.11–6.79; *p* < 0.001) while the risk of their middle-aged counterparts (OR = 1.23, 95% CI: 0.79–1.91; *p* = 0.317) did not differ from that of middle-aged never alcohol consumers. However, no significant difference was found in the increase in risk associated with alcohol consumption history between the middle-aged and elderly populations. These results are summarized in Table 15.

**Table 15.** **Comparisons of alcohol consumption history-associated risk of presence of AAC between middle-aged and elderly populations**.

Population	Regression coefficient^*^	OR^*^ (95% CI)^*^	*p* value^*^	*p* value for interaction^*^	*p* value for GOF
Middle-aged never alcohol consumers	Reference group			0.286	0.253
Middle-aged ever alcohol consumers	0.21	1.23 (0.79, 1.91)	0.317		
Elderly ever alcohol consumers	1.33	3.78 (2.11, 6.79)	<0.001		

Note: ^*^adjusting for sex, diabetes, hypertension, smoking history, coronary heart disease, fasting low-density lipoprotein cholesterol and usage of lipid-lowering agents. Abbreviations: AAC, abdominal aortic calcification; CI, confidence interval; GOF, goodness-of-fit; OR, odds ratio.

The result of further analysis on current alcohol consumption-associated risk of presence of AAC is summarized in Table 16. Elderly current alcohol consumers faced a significantly increased risk of AAC compared to that of middle-aged current non-alcohol consumers (OR = 3.62, 95% CI: 2.19–5.98; *p* < 0.001). Meanwhile, the risk among middle-aged current alcohol consumers was not significantly different from their non-consuming counterparts (OR = 1.31, 95% CI: 0.88–1.95; *p* = 0.170). Despite these variations, no statistically significant difference was observed in the elevation in AAC risk between middle-aged and elderly current alcohol consumers.

**Table 16.** **Comparisons of current alcohol consumption-associated risk of presence of AAC between middle-aged and elderly populations**.

Population	Regression coefficient^*^	OR^*^ (95% CI)^*^	*p* value^*^	*p* value for interaction^*^	*p* value for GOF
Middle-aged current non-alcohol consumers	Reference group			0.097	0.430
Middle-aged current alcohol consumers	0.27	1.31 (0.88, 1.95)	0.170		
Elderly current alcohol consumers	1.29	3.62 (2.19, 5.98)	<0.001		

Note: ^*^adjusting for sex, diabetes, hypertension, smoking history, coronary heart disease, fasting low-density lipoprotein cholesterol and usage of lipid-lowering agents. Abbreviations: AAC, abdominal aortic calcification; CI, confidence interval; GOF, goodness-of-fit; OR, odds ratio.

### 3.10 Multivariable Analyses of the Association Between Smoking History and Current Alcohol Consumption with the Presence of AAC or Severe AAC

Significant findings were noted in the multivariable analyses exploring the association of smoking history and current alcohol consumption with the presence and severity of AAC whose results were comprehensively adjusted for age, sex, diabetes, coronary heart disease, hypertension, and fasting LDL-C and are provided in Table 17.

**Table 17.** **Multivariable analysis of the association of smoking and alcohol consumption on presence of AAC**.

Variable	Regression coefficient	OR (95% CI)	*p* value	*p* value for GOF
Smoking history	0.50	1.65 (1.20, 2.27)	0.005	0.115
Current alcohol consumption	0.07	1.08 (0.82, 1.41)	0.569	
Male sex	0.03	1.03 (0.83, 1.28)	0.799	
Age ≥65	1.17	3.24 (2.31, 4.54)	<0.001	
CHD	0.32	1.38 (0.99, 1.93)	0.059	
Hypertension	0.48	1.61 (1.23, 2.11)	0.002	
Diabetes	0.17	1.18 (0.93, 1.51)	0.162	
Fasting LDL-C	0.01	1.01 (0.87, 1.17)	0.902	
Lipid-lowering agents	0.14	1.15 (0.88, 1.50)	0.292	

Abbreviations: AAC, abdominal aortic calcification; CHD, coronary heart disease; CI, confidence interval; GOF, goodness-of-fit; LDL-C, low-density lipoprotein cholesterol; OR, odds ratio.

After adjustment, smoking history showed a significant association with presence of AAC (OR = 1.65, 95% CI: 1.20–2.27; *p* = 0.005). By contrast, the association between current alcohol consumption and the presence of AAC was not significant (OR = 1.08, 95% CI: 0.82–1.41; *p* = 0.569). Additional findings highlighted the significance of elderly age (age ≥65) (OR = 3.24, 95% CI: 2.31–4.54; *p* < 0.001) and hypertension (OR = 1.61, 95% CI: 1.23–2.11; *p* = 0.002). The Archer-Lemeshow test offered no evidence for lack of fit of the model (*p* = 0.115), offering evidence of the validity of these results.

Similarly, as is shown in Table 18, smoking history was significantly associated with severe AAC (OR = 1.70, 95% CI: 1.26–2.29; *p* < 0.001). However, current alcohol consumption did not significantly correlated with the risk of severe AAC (OR = 1.03, 95% CI: 0.79–1.34; *p* = 0.800). Additional factors reaching statistical significance included age ≥65 (OR = 3.69, 95% CI: 2.66–5.10; *p* < 0.001), coronary heart disease (CHD) (OR = 1.72, 95% CI: 1.15–2.58; *p* = 0.013), and hypertension (OR = 1.61, 95% CI: 1.24–2.07; *p* < 0.001).

**Table 18.** **Multivariable analysis of the association of smoking and alcohol consumption on severe AAC**.

Variable	Regression coefficient	OR (95% CI)	*p* value
Smoking history	0.53	1.70 (1.26, 2.29)	<0.001
Current alcohol consumption	0.03	1.03 (0.79, 1.34)	0.800
Male sex	–0.01	0.99 (0.79, 1.25)	0.962
Age ≥65	1.30	3.69 (2.66, 5.10)	<0.001
CHD	0.54	1.72 (1.15, 2.58)	0.013
Hypertension	0.47	1.61 (1.24, 2.07)	<0.001
Diabetes	0.24	1.27 (1.01, 1.59)	0.044
Fasting LDL-C	0.00	1.00 (0.86, 1.17)	0.959
Lipid-lowering agents	0.17	1.18 (0.91, 1.53)	0.185

Abbreviations: AAC, abdominal aortic calcification; CHD, coronary heart disease; CI, confidence interval; LDL-C, low-density lipoprotein cholesterol; OR, odds ratio.

## 4. Discussion

Vascular calcification is a heterogeneous disease comprised of various subtypes with distinctly different risk factors. Vascular calcification can be classified as (1) intimal calcification, which is closely associated with atherosclerosis; (2) medial calcification, which is closely related to declined renal function and diabetes, and (3) genetic calcification [8]. As the nomenclature suggests, genetic calcification is associated with genetic disorders such as pseudoxanthoma elasticum (PXE) and Marfan syndrome [8], whose incidence are generally minuscule. For instance, the incidence of PXE between January, 2011 to December, 2020 stood at a mere 0.08 per 100,000 person-years [29]. Given the low prevalence of genetic disorders associated with genetic calcification, risk factors for intimal (atherosclerosis) and medial (renal function impairment and diabetes) are the major risk factors of AAC in the general middle-aged and elderly population. This is supported by findings from our study which observed an increase in AAC scores associated with advancing age and a decrease in eGFR. Similar conclusions concerning the prevalence of other AAC risk factors are also drawn, including smoking history as well as hypertension and diabetes diagnosis. These results reaffirmed the close association of AAC and atherosclerosis as well as the close relationship of traditional cardiovascular risk factors and AAC. However, the proportion of current smokers did not vary significantly across the groups. This, as is verified in the present study, is associated with the heterogeneity of risks of AAC within the subpopulation of current non-smokers, which is discussed later in this section.

The biological pathways through which smoking contributes to atherosclerosis and other cardiovascular diseases involve a highly complex mixture of compounds found in cigarette fumes and their interaction with the smoker’s personal traits, including environmental and genetic factors [30]. Endothelial dysfunction, a widely and long-lastingly studied topic, has been recognized as a pivotal mechanism associated with smoking-induced atherosclerosis. In as early as 1992, Celermajer *et al*. [31] found clinical evidence linking smoking to endothelial dysfunction as measured by decreased flow-mediated dilation. Subsequent biological studies have provided further insight into this phenomenon. Notably, nicotine has been shown to suppress endothelial nitric oxide synthase activation, leading to reduced nitric oxide production and increased nitric oxide depletion, culminating in endothelial and vascular dysfunction along with associated vascular calcification [32]. In addition, nicotine induces the production of inflammatory cytokines including interleukin (IL)-1β and IL-18 in human aortic endothelial cells, prompting vascular inflammation [33], another key mechanism of atherosclerosis. A recent study also revealed the role of oxidative stress and the release of extracellular vesicles in the development of nicotine-induced vascular calcification [34].

Clinical studies have also revealed the link between smoking and AAC. Jung *et al*. [9] analyzed risk factors for AAC in a cohort of 218 middle-aged and elderly men, identifying links between AAC and smoking-related indices, including cumulative smoking time and cumulative smoking amount. Similarly, Lioufas *et al*. [10] found a correlation between smoking and AAC in a cohort of 278 CKD patients. Extending these observations, this study utilized a larger and more representative sample drawn from a nationwide survey that was not restricted to sex or disease, aligning with previous research that underscored the association between smoking and AAC.

Results of this study indicate that the risks to which current smokers were exposed did not differ significantly from those of current non-smokers in terms of both the presence of AAC and severe AAC. From this, a hypothesis that those former smokers still faced a higher risk of AAC arose. If current non-smokers (both former smokers and never smokers) truly benefited from a lower risk of AAC, they should have been more prevalent in the group with an AAC score of 0 or the one with an AAC score between 0 and 6. In other words, the proportion of current non-smokers in the two groups should had been higher, while the proportion of current non-smokers in the group with an AAC score not less than 6 should had been lower. However, findings from the data contradicted this scenario, suggesting that the hypothesis of a homogeneous benefit among all current non-smokers might be incorrect. Further analysis comparing the risk of AAC among never, former, and current smokers confirmed this corollary. This finding is consistent with the research of Lv *et al*. [21], who analyzed the risk factors of severe AAC in the NHANES population using an identical definition of severe AAC as the one adopted by the current study. Their results confirmed a significantly increased risk of severe AAC for both former and current smokers compared to never smokers. Although our results parallel these findings in the sense of also revealing the association of AAC and smoking, a common atherosclerotic risk factor, as well as the cardiovascular harm of smoking, Lv *et al*. [21] found no significant difference in risk of severe AAC between former and current smokers while it was found in the present study that former smokers were benefited from a decrease in risk of presence of AAC compared to the currently smoking population. Taken together, these results suggest that the cardiovascular benefits associated with smoking cessation primarily manifest as reduction in the risk of AAC presence rather than severe AAC. Given the consistently elevated risks of AAC presence in elderly ever and current smokers revealed by subgroup analyses, promoting smoking cessation is of significant clinical and epidemiological importance, particularly for elderly individuals. This emphasizes the critical need for tobacco control measures targeting this population to mitigate their elevated risk of AAC and their associated cardiovascular complications.

The potential roles of alcoholic drinks, including ethanol and its coexistent compounds, in cardiovascular health are complex and not fully understood. From a biological perspective, alcohol affects the development of atherosclerosis in many respects. Alterations of blood lipid profile is one them. Alcohol has been shown to elevate high-density lipoprotein (HDL) levels except in individuals with severe hepatic dysfunction [35] and affect low-density lipoprotein (LDL) levels. However, the exact impact of alcohol consumption on LDL varies among different populations and is influenced by genetic variations [36, 37] and consumption patterns, such as binge versus regular drinking [35]. This contributes to the inconsistencies in the observed associations between alcohol consumption and atherosclerotic lesions like AAC.

Alcohol has also been found to modulate inflammatory mediators. Particularly, ethanol and non-alcoholic components of alcoholic drinks, such as polyphenols, downregulate the expression of intercellular adhesion molecule-1 (ICAM-1), vascular cell adhesion molecule-1 (VCAM-1) and E-selectin while also reducing monocyte adhesion to the vascular endothelium [38]. Despite these findings, evidence is sparse regarding the mechanisms via which excessive alcohol consumption leads to atherosclerotic lesions like AAC. This gap was noted by the finding in the subgroup of our study’s alcohol consumers who consumed more than two drinks per day over the past 12 months.

It has been postulated that the duo of ethanol and its metabolite, acetaldehyde, is crucial in determining the shift from atheroprotective to atherogenic effects of alcohol consumption [39]. More specifically, acetaldehyde has been shown to enhance monocyte adhesion to the endothelium, contributing to atherogenesis [40]. Once the level of acetaldehyde surpasses a certain threshold where the atherogenic effect of acetaldehyde balances the atheroprotective effect of ethanol, the effect of the former becomes dominant [39]. The interplay between alcohol and the elevation of blood pressure it induces [41] is another proposed conjecture of this transition where the detrimental effects of increased blood pressure may outweigh the cardiovascular benefits alcohol might provide [39].

Given the complexity of the biological actions of ethanol and other chemicals in alcoholic beverages, it is no surprise that clinical studies conducted thus far have reached inconsistent results. For example, Forbang *et al*. [13] observed a significant association between alcohol consumption and an increase in the natural logarithm of AAC volume among 1413 community-dwelling adult individuals with an AAC score above 0. However, Wu [14] found no association between alcohol consumption history and AAC in a Chinese cohort of patients with CKD stages 3–5. Similarly, this study found no association between alcohol consumption history and either the presence of AAC or severe AAC after adjusting for confounders in the entire cohort, which is in line with Wu’s results.

However, discrepancy between our results and those reported by Forbang *et al*. [13] exists. This may be attributed to several factors. First is measurement variability. AAC score assessment was conducted by different raters (radiologists or clinical doctors inspecting the radiographs and calculating AAC scores), which may introduce inter-observer variability. Second, AAC scores gauged the severity of AAC in terms of the extent of abdominal aorta afflicted. However, AAC may also be concentrated in one or more specific arterial segments and hence may not be perfectly correlated with overall extension. Third is the possibility of population differences. For example, the study by Forbang *et al*. [13] comprised mainly U.S. residents that included some individuals with Chinese ancestry, contrasting with Wu’s study [14] which focused on hospitalized patients in central China. These demographic and clinical differences can significantly impact the outcomes of studies examining the associations of alcohol and AAC.

In a more detailed analyses in the present study where the entire study population was grouped into never, former and current alcohol consumers to investigate the association of alcohol consumption with the presence of AAC, results indicated that neither former nor current alcohol consumers experienced significantly different risk of AAC compared to never alcohol consumers after adjusting for confounders. This finding aligns with the one obtained by McClelland *et al*. [42], who investigated the association of alcohol consumption with coronary artery calcification in an American population and also found no significant differences among never, former, and current alcohol consumers. This consistency may be attributed to two factors. First, vascular calcification in different arterial segments share a common taxonomy (i.e., intimal, medial and genetic calcification), each associated with specific risk factors and potentially considered as manifestations of a single disease in different locations. Second, both the current study and the study by McClelland *et al*. [42] involved American, middle-aged and elderly populations (investigated subjects were ≥40 years old in the present study while the ones in McClelland *et al*. [42] aged between 45 and 84 years old), which may suggest a geographic or demographic consistency in these findings. However, the study by McClelland *et al*. [42] did not involve age subgroup analyses while it was found in this study that elderly ever and current alcohol consumers were both exposed to a significantly elevated risk of presence of AAC.

The results discussed above were obtained from both univariable and multivariable analyses, with confounders fully adjusted for in the latter. A confounder is defined by three essential characteristics: (1) it is associated with the exposure, (2) it is related to the outcome, and (3) it is not an intermediary in the causal pathway between the exposure and the outcome [43]. Based on these criteria, this study carefully selected several variables as confounders for adjustment: sex, age, diabetes, hypertension, CHD, LDL-C, and usage of lipid-lowering agents. The following paragraphs outline evidence in establishing the association of the confounder and the outcome (i.e., AAC) as well as the exposures (i.e., smoking and alcohol consumption), justifying their role as confounders adjusted in the analyses.

(1) Sex plays ubiquitous roles in various physiological and pathophysiological conditions, with vascular calcification being no exception. Males typically exhibit an increased prevalence of vascular calcification compared to females [44]. Additionally, significant difference in the proportions of smokers between males and females have been observed, highlighting the association between sex and the risk factor [45]. Furthermore, there is also a notable sex difference in alcohol consumption patterns, specifically in the mean number of standard drinks consumed, which has been found to vary significantly between males and females, providing evidence for the association between sex and alcohol consumption [46].

(2) Age is a well-recognized risk factor for atherosclerosis and vascular calcification. Age has been shown to be significantly associated with calcification in both the coronary artery and the aorta, underscoring its influence on vascular health across different body regions [47]. Additionally, age has been shown to be statistically significantly different among never, former and current smokers [11]. Moreover, an age-associated increase in the mean number of standard drinks consumed has been observed [46].

(3) Diabetes is intricately linked with vascular calcification, a relationship that has been emphasized early in this discussion. Moreover, there is a documented correlation between smoking and diabetes [48]. Additionally, the consumption of alcohol, specifically at low and moderate levels, has also been found to be associated with diabetes [49].

(4) Hypertension is significantly correlated with vascular calcification [50]. Furthermore, smoking is recognized as a risk factor for hypertension, establishing a direct link between the risk factor and the disease [51]. Additionally, the proportion of hypertensive individuals increases with the amount of alcohol consumed. This observation underscores the association between hypertension and alcohol consumption [42].

(5) Atherosclerosis, as mentioned above, has close connections to vascular calcification. Coronary artery calcification has been shown to be associated with AAC [52], underscoring the shared pathological processes between these conditions and the link between coronary atherosclerosis and AAC. Moreover, smoking is a widely recognized risk factor for CHD [53]. Additionally, there is a documented association between alcohol consumption and CHD [54].

(6) The accumulation of LDL-C is one of the first steps in atherosclerosis [1]. Smoking has been found to be associated with alterations in LDL-C concentrations [55]. Research has indicated a lack of association between LDL-C and alcohol consumption [42]. However, there is evidence suggesting an association between the ratio of LDL-C and high-density lipoprotein cholesterol (HDL-C) and alcohol consumption [56].

(7) Usage of lipid-lowering agents, particularly statins, has been associated with vascular calcification [57]. When examining the connection between statin use and smoking, the available evidence presents a mixed picture. A research project reported no direct association between statin use and smoking [58], while another one observed a noticeable difference in smoking prevalence—approximately 10%—between statin users and non-users [59]. The relationship of alcohol consumption and statin use is similarly complex. While one study found little difference in the proportions of various categories of daily alcohol consumption between statin users and non-users [60], another documented a statistically significant association between alcohol consumption and usage of lipid-lowering agents [61]. Taken together, it is decided to accept lipid-lowering agents as a confounder to adjust for in the analyses.

Despite rigorous attempts, the current study is subject to several limitations that may impact the validity of its findings. The first is the risk of recall bias. Histories of smoking and alcohol consumption relied on self- or proxy-reported information. This method is prone to recall bias, where respondents may not accurately remember or may choose to selectively report their consumption habits. The second is the problem of inter-observer variability. The AAC score was based on radiograph inspection. Different observers may interpret radiographic images differently, leading to inconsistencies in the AAC scores calculated. A third limitation regards the AAC score itself, which quantifies disease severity by examining its extent. However, when AAC is localized and not widely distributed, the AAC score may not accurately reflect the true severity of the condition. Fourth is the possibility of residual confounding variables. While efforts were made to control for confounders, the possibility of residual confounding variables remain. For instance, the NHANES dataset does not include information on certain comorbidities like systemic lupus erythematosus, which could influence the results. These limitations necessitate cautious interpretation of the study’s results and suggest that future research should consider more robust methods for data collection and analysis to mitigate these issues.

Given the above limitations, we propose a series of proactive steps that can be used to mitigate similar limitations in future studies. The implementation of standardized training and operating procedures for AAC raters should be employed to limit measurement errors and inter-observer variability. Consistent systematic training ensures that all raters apply the same criteria when evaluating radiographs, leading to more reliable and comparable results across various studies. Modern medicine is increasingly integrating artificial intelligence (AI) technology. This includes utilizing AI tools which can significantly enhance the accuracy and consistency of measurements. AI algorithms can analyze imaging data with high precision and less bias, providing a standardized assessment of calcification that is not influenced by human error. To address recall bias, the use medical insurance reimbursement data and medical records can serve as an effective method for cross-validating responses to the survey questions. By cross-referencing participants’ responses with their medical records, researchers can confirm the accuracy of reported smoking and alcohol consumption habits and other relevant health information. This approach helps enhance the reliability of the data and strengthens the study’s findings.

From a statistical and mathematical perspective, diagnosis and treatment in clinical care, two essentials of everyday clinical practice, can be conceptualized as classification and decision-making tasks, respectively. These tasks align closely with areas extensively explored within mathematics and statistics. For instance, as research continues to reveal the heterogeneity in the associations of alcohol consumption on AAC across different populations, such as those with varying genotypes, there is an emerging opportunity to apply statistical decision-theoretic methods. These methods could quantitatively assess the benefits and risks of alcohol consumption, supporting the development of decision support systems incorporated as modules within hospital information systems (HIS). Such systems or modules would enable health care professionals to readily formulate personalized responses to the question of “Is alcohol good for my health?” in the outpatient clinic and formulate practical guidance on alcohol consumption (e.g., amount, frequency or type of alcohol intake that ensures enhancement of health) or even “alcohol prescriptions” tailored for each patient. Additionally, mathematical models can be valuable in simulating the microenvironment of cigarette fume- or ethanol-exposed endothelium. These models offer not only a dynamic description of pathophysiological processes but also a quantitative framework for understanding these biological interactions more deeply than qualitative descriptions alone. Given the practical challenges in replicating prolonged exposure to cigarette or alcohol in humans through animal, cell, and other biological studies, mathematical modeling presents a promising alternative for quantifying these long-term biological effects. The integration of interdisciplinary scientific approaches promises to enhance our understanding of human health and disease comprehensively. In line with the principles of precision and evidence-based medicine, this interdisciplinary approach could herald the advent of the epoch of algorithm-supported healthcare.

## 5. Conclusions

Using nationwide survey data, the current study identifies a significant association between smoking history and both the presence of AAC and severe AAC, whereas current smoking shows no such association. Notably, compared to never smokers, former smokers are at a significantly higher risk for AAC, yet their risk remain lower than that of current smokers. Subgroup analyses identify elderly ever and current smokers as victims of increased AAC risk, as do middle-aged ever smokers. These results indicate that smoking is an independent risk factor for AAC, with stronger risk in the elderly population. Results of this study and the ones obtained by previously conducted studies collectively indicate that the cardiovascular benefits associated with smoking cessation primarily manifest as reduction in the risk of AAC presence rather than severe AAC.

By contrast, the study found no significant associations between either ever or current alcohol consumption and AAC or severe AAC in the entire cohort after adjusting for confounders. Compared with never alcohol consumers, AAC risk do not significantly differ among former or current alcohol consumers. However, subjects consuming more than 2 drinks of alcohol per day as well as the elderly ever and current alcohol consumers suffer from increased AAC risk. These results indicate that alcohol consumption is not an independent risk factor of AAC except in the heavy drinking and elderly subpopulations.

## Availability of Data and Materials

This study utilizes data of NHANES 2013-2014, a publicly available dataset that can be downloaded from its website: https://wwwn.cdc.gov/nchs/nhanes/continuousnhanes/default.aspx?BeginYear=2013. All data this study used can be directly downloaded there.
